# Root adaptations to soils with low fertility and aluminium toxicity

**DOI:** 10.1093/aob/mcw073

**Published:** 2016-06-01

**Authors:** Idupulapati M. Rao, John W. Miles, Stephen E. Beebe, Walter J. Horst

**Affiliations:** ^1^Centro Internacional de Agricultura Tropical (CIAT), A. A. 6713, Cali, Colombia and; ^2^Leibniz University of Hannover, Herrenhaeuser Str. 2, D-30419 Hannover, Germany

**Keywords:** aluminium, breeding, interspecific hybridization, low soil fertility, nitrogen, nutrient acquisition, phosphorus, problem soils, root phenes, root physiology

## Abstract

**Background** Plants depend on their root systems to acquire the water and nutrients necessary for their survival in nature, and for their yield and nutritional quality in agriculture. Root systems are complex and a variety of root phenes have been identified as contributors to adaptation to soils with low fertility and aluminium (Al) toxicity. Phenotypic characterization of root adaptations to infertile soils is enabling plant breeders to develop improved cultivars that not only yield more, but also contribute to yield stability and nutritional security in the face of climate variability.

**Scope** In this review the adaptive responses of root systems to soils with low fertility and Al toxicity are described. After a brief introduction, the purpose and focus of the review are outlined. This is followed by a description of the adaptive responses of roots to low supply of mineral nutrients [with an emphasis on low availability of nitrogen (N) and phosphorus (P) and on toxic levels of Al]. We describe progress in developing germplasm adapted to soils with low fertility or Al toxicity using selected examples from ongoing breeding programmes on food (maize, common bean) and forage/feed (*Brachiaria* spp.) crops. A number of root architectural, morphological, anatomical and metabolic phenes contribute to the superior performance and yield on soils with low fertility and Al toxicity. Major advances have been made in identifying root phenes in improving adaptation to low N (maize), low P (common bean) or high Al [maize, common bean, species and hybrids of brachiariagrass, bulbous canarygrass (*Phalaris aquatica*) and lucerne (*Medicago sativa*)].

**Conclusions** Advanced root phenotyping tools will allow dissection of root responses into specific root phenes that will aid both conventional and molecular breeders to develop superior cultivars. These new cultivars will play a key role in sustainable intensification of crop–livestock systems, particularly in smallholder systems of the tropics. Development of these new cultivars adapted to soils with low fertility and Al toxicity is needed to improve global food and nutritional security and environmental sustainability.

## INTRODUCTION

Plants colonized the land around 450 to 490 Mya (Dolan, 2009). This required several adaptations, including the uptake and movement of water and solutes within the plant (Pires and Dolan, 2012). Roots are multicellular organs characterized by particular features such as gravitropic response, endogenous branching, root hairs and a protective root cap (Kenrick and Strullu-Derrien, 2014). The evolution of roots served plants for a wide variety of processes, including nutrient and water uptake, anchoring and mechanical support, storage functions, and as the major interface between the plant and various biotic and abiotic factors in the soil environment (De Smet *et al.*, 2012; Tian *et al.*, 2014). Thus, plants depend on their root systems for their survival in nature, and for their yield and nutritional quality in agriculture. Root systems are complex and a variety of traits have been identified over the past decade as contributing to adaptation to low fertility and/or toxic soils. Plant root systems comprise a set of phenes, or traits, that interact with the environment, and phenes are the identifiable units of the plant phenotype (York *et al.*, 2013). Phenotypic characterization of root adaptations to soils with low fertility and aluminium (Al) toxicity is enabling plant breeders to develop cultivars that not only yield more, but also contribute to yield stability and nutritional security in the face of climate variability.

Food insecurity is among the greatest challenges that humanity is facing in the 21^st^ century. Low soil fertility including low availability of nutrients and problems such as soil acidity and associated Al toxicity limits agricultural productivity by restraining crops from reaching their yield potential. Excessive use of fertilizer in developed countries, including China, pollutes the air and water and contributes to climate change and environmental degradation. Low soil fertility in developing countries is a primary constraint to food security and economic development. Additionally, water availability often limits crop growth in most agricultural systems. Increasing the efficiency of plants in acquiring soil resources is a key approach to improve crop yields and to reduce the dependence of farmers on fertilizers or irrigation (Bishopp and Lynch, 2015).

Soil-related constraints [mainly nutrient deficiencies particularly of nitrogen (N) and phosphorus (P), soil acidity-related Al toxicity, and salinity] and insufficient water availability are probably the biggest cause of a persistent gap between potential and realized crop productivity, particularly in developing countries in the tropics. The nutrients that soils naturally supply to plants come from the dissolution of primary and particularly weatherable minerals. About 36 % of the tropics has soils with low (<10 %) reserves of weatherable minerals in their sand and silt fraction, which constitute nutrient capital reserves (Sánchez-Calderón *et al.*, 2013; Table 1). The proportion of nutrients held in soil organic matter influences these deficiencies.

**Table 1.** Main chemical constraints relevant to agriculture in the tropics (adapted from Sanchez et al., 2003)

Soil chemical constraint	Million hectares	% of area affected*
Low nutrient reserves (<10 % weatherable minerals)	1681	36
Aluminum toxicity (>60 % Al saturation)	1493	32
No major chemical limitation (pH 5·5–7·2)	1198	26
High phosphorus fixation (by Fe and Al sesquioxides)	1065	23
Calcareous (micronutrient deficiencies)	152	3
Total	4639	

*The sum of percentages exceeds 100 because a single soil often has more than one attribute.

General symptoms of crop plants due to low soil fertility or mineral toxicity include poor emergence; slow growth; seedling and adult plant stunting; leaf chlorosis and bronzing; reduced overall growth and dry-matter production; delayed and prolonged flowering and maturity; flower and pod abortion; low harvest index; reduced seed weight; deformed and discoloured seeds; and severe yield loss. Root growth, development and distribution across the soil profile are adversely affected by soil chemical constraints (Marschner, 1995; Lynch, 1995; Gregory, 2006). Improved adaptation of a crop to infertile soils can be achieved by two general approaches: the growth environment may be altered, or the plant genotype may be improved. Often a combined approach is the most effective.

Plant adaptation to infertile soils has complex inheritance and is affected by the growing environment, and consequently the genetic and physiological mechanisms leading to improvements in adaptation have been difficult to identify and to quantify. However, understanding the mechanisms by which plants adapt to infertile soils is critical for creating efficient strategies to develop stress-resistant cultivars for the sustainable intensification of production systems.

The most successful approaches to improving crop adaptation to infertile soils have historically used field-based evaluations to identify tolerant genotypes, followed by breeding and selection of cultivars that combine performance in stressful environments with other desirable plant attributes. One aspect of germplasm improvement is to identify morphological, physiological and biochemical mechanisms by which plants adapt to soils with low fertility and Al toxicity. Defining specific mechanisms of adaptation to these soil constraints can contribute to the development of high-throughput phenotyping protocols improving the efficiency of genetic improvement programmes.

Three major soil constraints for crop and forage production in developing countries are low N and P availability and soil acidity-induced Al toxicity. Soil nutrients can be relatively mobile or immobile (Barber, 1995). Nitrogen in the form of nitrate and sulphur (S) in the form of sulphate are highly mobile, whereas P is the most immobile macronutrient. Potassium (K) and ammonium-N are also relatively immobile, as are most micronutrients, whereas calcium (Ca) and magnesium (Mg) have intermediate mobility. Diffusion is important particularly for acquisition of P and K, while mass flow is more important for acquisition of all other macro- and micronutrients (White *et al.*, 2013*a*). The acquisition of different soil nutrients, and often different chemical forms of mineral elements, requires different root adaptations. Toxicity of Al and deficiency of P tend to occur in parallel in low fertility acid soils (Marschner, 1995). The ability to resist toxic mineral elements such as Al also requires different root adaptations.

The phenotype of an organism is fundamentally a manifestation of its genotype’s interaction with its environment (Cobb *et al.*, 2013). Many common measures of root system and individual root properties are examples of phene aggregates that are influenced by several, more elemental root phenes, and some are partially functional responses dependent on plant performance. York *et al.* (2013) defined these root measurements and the phenes that influence each root measurement. For example, the measurement of total root length or root length density is influenced by axial root length, number of axial roots, lateral branching and lateral length while specific root length is influenced by xylem area, phloem area, number and size of cortical cells, aerenchyma area, diameter distribution and secondary root development. Rooting depth is influenced by axial root angles, axial root length, axial root number, lateral root branching and lateral root length. De Smet *et al.* (2012) commented on assays to describe lateral root phenotypes and proposed ways to advance the description of root system architecture.

Several authors have reviewed research efforts on adaptation of crops to soils of low fertility and toxicities in a general sense (Marschner, 1991; Lynch and St. Clair, 2004; George *et al.*, 2012; Lynch and Brown, 2012; Araújo *et al.*, 2015). Others have focused on specific single constraints such as low P (Lynch and Beebe, 1995; Lambers *et al.*, 1998; Ramaekers *et al.*, 2010; Lynch, 2011; Richardson *et al.*, 2011; Veneklaas *et al.*, 2012; Brown *et al.*, 2013; Zhang *et al.*, 2014), low N (Kraiser *et al.*, 2011; Lynch, 2013; Niu *et al.*, 2013), Al toxicity (Kochian *et al.*, 2004; Horst *et al.*, 2010; Yang *et al.*, 2013) or the specific role of root characteristics in adaptation (Lynch, 2007, 2011; Kell, 2011; Jung and McCouch, 2013; White *et al.*, 2013*a*, *b*; Kudoyarova *et al.*, 2015; Paez-Garcia *et al.*, 2015).

The aim of this paper is to review the state of our understanding of the adaptive responses of root systems to soils with low fertility focusing on P and N limitations and soil acidity-related Al toxicity with the objective to develop strategies for the breeding of crops to acid, Al-toxic soils with low fertility with complex soil-related constraints. We first describe the adaptive responses of roots to low supply of mineral elements (with a particular emphasis on low N and P availability in soil) and toxic level of Al in soil. We then discuss the progress in developing germplasm adapted to soils with low fertility and Al toxicity using examples from ongoing breeding programmes. The case studies selected include two major food crops, maize (*Zea mays* L.) and common bean (*Phaseolus vulgaris* L.) and one major forage/feed crop, brachiaria (*Brachiaria* spp.). We highlight the importance of developing a robust understanding of root adaptations to design superior root ideotypes to match the environment. We conclude by discussing the challenges and opportunities for 21^st^-century breeding to design soil stress-resilient crop and forage cultivars for sustainable intensification of crop–livestock systems leading to improved food and nutritional security and environmental sustainability.

## ROOT SYSTEM RESPONSES TO LOW-FERTILITY SOILS

Roots of angiosperms can be classified into embryonic or post-embryonic (Atkinson *et al.*, 2014). Post-embryonic roots arising from tissues other than roots are termed adventitious. Root systems based on development of the primary embryonic root of dicots are also known as taproot or allorhizic root systems and those in monocots that are composed mostly of adventitious roots are termed fibrous or homorhizic root systems. In cereal crops the majority of the mature root system is composed of several classes of adventitious roots that include crown roots and brace roots.

Higher plants present a wide diversity of root system architectures (RSAs; spatial configuration of the root system) among species for effective performance under low fertility soil conditions (Lynch, 1995). Each kind of RSA is guided by a genetically controlled, so-called ‘postembrionary root developmental programme’ (PERDP), which is not deterministic, and permits phenotypic plasticity in response to environmental conditions, including the availability of nutrients (Sánchez-Calderón *et al.*, 2013). PERDP is essentially driven by two cellular processes: (1) cell division in the apical root meristem and new lateral meristems formed from the pericycle; and (2) cell expansion occurring in the root elongation zone. This particular characteristic permits plants, which are sessile organisms, to change their root architecture to adapt to changing soil fertility (López-Bucio *et al.*, 2003; Hodge, 2004; Hodge *et al.*, 2009).

Plant root adaptive growth in response to low availability of macro- and micronutrients depends on a wide range of variables such as nutrient forms, availability, concentration, localization and nutrient behaviour in soil, as well as the nutrient status of the plant (Jung and McCouch, 2013). Root growth in response to a nutrient stimulus requires four main steps: stimulus perception, signal transduction, target gene regulation and gene product mediation of growth response. Root architectural traits, including the number, length, orientation and branching of several root classes, contribute to the superior performance and yield of crops grown on low-fertility soils (Lynch, 2011). These are central to resource acquisition from low-fertility soils (York *et al.*, 2013).

A number of root system responses contribute to plant adaptation to low-fertility soils (Table 2; White *et al.*, 2013*b*). These include the ability to: (1) increase the volume of soil explored by the root system and the root surface area for the uptake of nutrients (root elongation rate, lateral root production, root hair characteristics, root length density, ability to penetrate soil); (2) exploit different soil horizons (gravitropism of root growth); (3) reduce carbon and energy requirement for nutrient acquisition (the proliferation of roots in patches of soil containing high concentration of nutrients that are immobile in soil); (4) increase fine root turnover to redistribute carbon following the capture of localized nutrients; (5) reduce root respiration through increased specific root length and formation of aerenchyma; (6) develop high-capacity nutrient uptake systems for elements whose delivery to the root surface is determined by diffusion in the rhizosphere; (7) affect the concentration of nutrients in the soil solution either directly through soil chemistry or indirectly through colonization by appropriate microbial communities (modification of rhizosphere pH, exudation of organic solutes and enzymes); and (8) interact with microbes either intimately, through mycorrhizal associations or nodulation, or remotely, through facilitating the colonization by beneficial microbes or exclusion of pathogenic organisms in the rhizosphere. A number of these root morphological and physiological responses to low soil fertility (nutrient deficiencies) are regulated by plant hormones (Kudoyarova *et al.*, 2015) and particularly ethylene (García *et al.*, 2015). Root exudates are considered as key players in the selection of the microbial community during plant–microbe interactions that impact plant productivity in the field (De-la-Peña and Loyola-Vargas, 2014).

**Table 2.** Root adaptive traits/phenes to soils with low availability of nitrogen and phosphorus and toxic level of aluminium (modified from Paez-Garcia et al., 2015)

Soil constraints	Root traits/phenes	Description	Reference(s)
Low nitrogen	Rooting depth	A high rate of nitrate supply inhibits rooting depth in some soils.	Forde (2014)
	Root hairs	High nitrate reduces root hair length in some plant species.	
	Root branching	External nitrate stimulates lateral root initiation and elongation, whereas a high plant internal nitrate/N status inhibits lateral root growth. Early lateral root development can be inhibited. Reduced frequency of lateral root branching and longer lateral roots improve N capture from low-N soils.	Forde (2014), Zhan and Lynch (2015), Walch-Liu *et al.* (2006)
		Plants with brace and crown roots growing at shallower angle are more N efficient. Reduced crown root number is associated with greater rooting depth, N capture and yield.	York *et al.* (2015), Lynch (2015), Saengwilai *et al.* (2014*a*)
	Anatomical root traits	Root cortical aerenchyma (RCA) formation is induced to reduce respiration, N content of root tissue and the metabolic cost of soil exploration. RCA formation increases rooting depth, N capture and biomass/yield.	Drew *et al.* (1989), Postma and Lynch (2011), Lynch (2015), Saengwilai *et al.* (2014*b*), York *et al.* (2015)
	Metabolic root traits	Decreased specific root respiration due to decreased cortical cell number and size and increased cortical aerenchyma.	Lynch (2015)
Low phosphorus	Rooting depth	Primary root growth is inhibited.	López-Bucio *et al.* (2003)
	Root hairs	Proliferation of root hairs is stimulated, root hairs can contribute 70 % or more of the total root surface area and can be responsible for up to 90 % of P acquired.	Bates and Lynch (2001), Haling *et al.* (2013), Miguel *et al.* (2015)
	Root branching	Lateral root initiation and emergence is stimulated. A reduced gravitropic trajectory of basal roots, adventitious rooting and altered dispersion of lateral roots enable topsoil foraging in response. Shallow basal roots improve P acquisition in the field.	Den Herder *et al.* (2010), Postma *et al.* (2014), Lynch and Brown (2001), Miguel *et al.* (2015), Mori *et al.* (2016)
		Cluster roots are better able to access P by producing large amounts of exudates containing phosphatases and carboxylates that help release bound P.	Neumann and Martinoia (2002), Lambers *et al.* (2011)
	Anatomical root traits	Root cortical aerenchyma formation is induced to reduce respiration, P content of root tissue and metabolic cost of soil exploration.	Drew *et al.* (1989), Postma and Lynch (2011), Lynch (2015), Lynch *et al.* (2014)
	Metabolic root traits	Reduced root respiration reduces the metabolic cost of soil exploration; increased production of carboxylates and phosphatases.	Nielsen *et al.* (1998, 2001), Lynch (2015)
High aluminium	Rooting depth	Root elongation is inhibited with swollen and malformed root tips	Horst *et al.* (1992), Delhaize *et al.* (1993), George *et al.* (2012)
	Root hairs	Rhizosheath presence is correlated with the Al tolerance of root hairs; deformed root hairs.	Delhaize *et al.* (2012)
	Root branching	Inhibition of lateral root initiation and outgrowth.	George *et al.* (2012), Jung and McCouch (2013)
	Anatomical root traits	Inhibition of cell expansion and cell division; change in cell patterning leading to stimulation of cell division in distal transition zone.	Yang *et al.* (2013), Kopittke *et al.* (2015)
	Metabolic root traits	Increased production of carboxylates; disruption of plasma membrane properties	Yang *et al.* (2013), Kochian *et al.* (2015)

Long-term research efforts to improve crop adaptation to low soil fertility in the tropics have been limited. Genetic improvement in crop adaptation to low fertility soils is, indeed, possible, albeit by ‘brute-force screening under stress’. Improving adaptation to low soil fertility has been identified as one of the high priorities for research on common bean improvement by several countries in Africa. Here we highlight the progress from these efforts. Common bean is the most important food legume and soil infertility is a major limitation for bean productivity by smallholder farmers in Africa (Beebe, 2012). Average yields are very low, ranging from 200 to 700 kg ha^−1^ and losses due to low soil fertility are estimated at over 1·12 million tonnes every year (Wortmann *et al.*, 1998). Considerable effort has been made over the last two decades by the East and Central Africa Bean Research Network (ECABREN) and the Southern Africa Bean Research Network (SABRN), both of which belong to the Pan Africa Bean Research Alliance (PABRA), to develop and promote low soil fertility-adapted bean varieties and soil management technologies that enhance resilience to soil constraints and boost bean productivity (Lunze *et al.*, 2011). A total of 1400 bean lines have been evaluated through BILFA (Bean Improvement for Low Fertility soils in Africa) for their relative tolerance to the stresses under consideration, particularly low N, low P and soil acidity with the associated Al and/or manganese (Mn) toxicities. Considerable genetic variability in germplasm was detected and several genotypes with specific single or multiple edaphic stress tolerance were identified. Field evaluation in multiple sites and countries resulted in identification of several bean lines tolerant to low soil fertility. These include five lines tolerant to low N, seven lines tolerant to low P, four lines tolerant to low K, two lines resistant to high Al and three lines resistant to high Mn. Of these selected lines, seven were released as low soil fertility-adapted varieties that were adopted by smallholders in seven countries (Lunze *et al.*, 2011). Breeding efforts may be made more effective and efficient with greater physiological understanding of the phenomena involved at root system level.

## ROOT ADAPTATIONS TO LOW NITROGEN SOILS

Nitrogen is the plant nutrient needed in largest quantities. An increase in N supply stimulates plant growth rates and biomass (Marschner, 1995). Low soil N availability is a major constraint to agricultural productivity in smallholder systems in developing countries, particularly in sub-Saharan Africa, where less than 20 kg N ha^−1^ is applied to fields due to high fertilizer costs (Azeez *et al.*, 2006; Worku *et al.*, 2007). This situation is in contrast to developed countries where intensive N fertilization is resulting in substantial environmental and economic costs (Sutton *et al.*, 2011). Kant *et al.* (2011) estimated that a 1 % increase in crop N acquisition and utilization efficiency (N efficiency) worldwide could save approximately US$1·1 billion annually. Ammonium and nitrate are two major soil N forms available to plants. Ammonium is the predominant form in acid soils while nitrate is the main N form in most crop production environments (Miller and Cramer, 2004). Nitrate is highly mobile compared with ammonium, and after rainfall or irrigation, nitrate is leached to deeper soil. Leaching of nitrate is the major cause of poor recovery of applied N fertilizer to crops. One of the key strategies to improve crop N acquisition efficiency is through selection of root phenes that enhance rapid deep soil exploration (Lynch, 2013). In low-N environments root growth is reduced less than shoot growth leading to an increased root to shoot ratio (Marschner, 1995). Whereas high N supply enhances root branching at the site of fertilizer-N application in the surface soil, rooting depth may be reduced compared to low N supply.

Crop N efficiency can be enhanced by increasing N acquisition through improved root traits and N utilization by shoot traits (Raun *et al.*, 2002; Kant *et al.*, 2011; Xu *et al.*, 2012), but the relevance of root phenes has received less attention than shoot phenes until recently (Table 2). Lynch (2013) proposed the ‘Steep, cheap and deep’ (SCD) root ideotype which consists of architectural, anatomical, morphological and physiological phenes that work together to improve the capture of water and N in leaching environments by accelerating subsoil exploration. Maize genotypes with few crown roots (crown root number, CN) were shown to have greater N acquisition from low-N soils (Saengwilai *et al.*, 2014*a*). Under low N conditions low CN genotypes acquired more N from deep soil strata than high CN genotypes, leading to greater photosynthesis and plant N content. Using recombinant inbred lines (RILs) of maize with the ability to form high root cortical aerenchyma (RCA), Saengwilai *et al.* (2014*b*) showed that RCA improves plant growth under low N conditions by decreasing root metabolic costs, thereby increasing soil exploration and N acquisition from deep soil strata. Zhan and Lynch (2015) showed that a few but long lateral roots in RILs of maize improve N capture from low N soils. Analysis of the evolution of root architectural and anatomical phenes of US maize over the past 100 years using 16 cultivars indicated increased tolerance of low N in modern varieties (York *et al.*, 2015). This study also showed that the evolution of maize root phenotypes over the past century is consistent with increasing N use efficiency (NUE). Manipulating genes regulating root growth and activity could improve NUE (Xu *et al.*, 2012).

In eastern and southern Africa, the IMAS (Improved Maize for African Soils) project (coordinated by Dr B. Das of CIMMYT) has established the world’s largest low-N screening network for maize, with 25 sites in ten countries and a total of over 120 000 experimental plots. In 2014, partners in the IMAS project developed 41 maize cultivars that respond better to low amounts of N fertilizer and these are due for release in nine African countries through 24 seed companies (http://blog.cimmyt.org/improved-maize-to-boost-yields-in-nitrogen-starved-african-soils/). Crucially for farmers, these varieties also perform well under well-fertilized conditions, whilst several carry resistance to maize lethal necrosis, a devastating viral disease spreading through eastern Africa. IMAS is aiming to raise maize yields by 50 % and benefit up to 60 million maize farmers in eastern and southern Africa. It will be a major challenge to quantify the relative contribution of root adaptations to low N stress and the improved yield of these varieties in farmers’ fields.

An innovative strategy to improve adaptation to low N soils and to increase NUE by crops is through biological nitrification inhibition (BNI) in soil (Subbarao *et al.*, 2015). Manipulation of the release of nitrification inhibitors from roots through root exudation and root turnover limits the amount of N cycled through the soil nitrification pathway and, thereby, improves NUE and minimizes N pollution from agricultural systems. There is extensive genetic variation in BNI both among and within plant species (Subbarao *et al.*, 2007), which may allow the breeding of crops with increased BNI to reduce the rate of ammonium oxidation, nitrate leaching and nitrous oxide emission from agricultural soils (Subbarao *et al.*, 2009).

## ROOT ADAPTATIONS TO LOW PHOSPHORUS SOILS

Phosphorus availability in soil limits plant growth and yield. Approximately half of the world’s agricultural lands are P-deficient (Fairhurst *et al.*, 1999; Lynch, 2011). Significant increases in P demand by plants are probable, owing to stimulation of photosynthesis under elevated carbon dioxide concentrations and consequent shoot and root growth responses (Jin *et al.*, 2015). Many P-deficient soils occur in developing countries, where they are often degraded and where farmers lack the financial resources to purchase P fertilizers. Compared with other major nutrients, P is by far the least mobile and the least available to plants in most soil conditions (Schachtman *et al.*, 1998; Hinsinger, 2001). Most soils that have little plant-available P contain considerable amounts of P, but a large proportion is bound to different soil constituents, forming complexes that limit availability (Driessen *et al.*, 2001; Kochian, 2012). These soils impose agronomic and economic constraints. Application of P fertilizer is common practice, and necessary if agricultural productivity is not to be seriously limited. Improving P fertilizer use efficiency of crops and forages through genetic adaptation to low to moderate levels of applied P is critical for smallholder agriculture (Lunze *et al.*, 2011; Beebe *et al.*, 2013). Improved cultivars with genetic adaptation to low-P soils may be a viable complement to P fertilization, particularly for crop–livestock systems (Niu *et al.*, 2013).

Plants possess several adaptive mechanisms to cope with P deficiency, including changes at the morphological, physiological, biochemical and molecular levels (Zhang *et al.*, 2014). These mechanisms result either in increased acquisition of P from the soil or in a more efficient internal use of P (Rao *et al.*, 1999; Vance *et al.*, 2003; Lynch, 2011; Richardson *et al.*, 2011; Veneklaas *et al.*, 2012). An effective management strategy for soils with low P content and/or P fixation is to enhance the plant’s efficiency in acquiring soil P (Lynch, 2011). Improved P acquisition by crop plants can be addressed through one or more of three approaches: (1) traditional plant breeding for enhanced P acquisition; (2) genetic engineering to introduce genes that improve P acquisition and growth of crop plants; and (3) inoculation with plant growth-promoting rhizobacteria and mycorrhizae (Lynch, 1995; Ramaekers *et al.*, 2010; Richardson and Simpson, 2011; Lynch, 2011; López-Arredondo *et al.*, 2014; Zhang *et al.*, 2014).

Long-term research using common bean has contributed to defining root phenes and their role in enhanced soil exploration and P acquisition (Table 2; Lynch, 2011). In common bean, basal root whorl number (BRWN) differs among genotypes from one to four, with each whorl typically generating four basal roots (Lynch, 2011). Uppermost whorls produce basal roots with shallower growth angle while lower whorls produce roots of progressively steeper angle. Greater BRWN could increase soil exploration by increasing the vertical range of root deployment (Lynch, 2011). One of the key mechanisms to increase access to P is greater *topsoil foraging* resulting from root architectural, morphological and anatomical traits (Lynch, 2011; Richardson and Simpson, 2011). The ideotype of *topsoil foraging* has been proposed for improving P acquisition efficiency (PAE) (Lynch and Brown, 2001; Lynch, 2011; White *et al.*, 2013*b*; Lynch and Wojciechowski, 2015). It incorporates: (1) early root vigour and preferential production of roots in topsoil; (2) greater root branching and the production of long root hairs; (3) high root length density in the topsoil and the proliferation of lateral roots in P-rich patches; (4) greater root length/mass quotient, either through the development of thinner roots or the formation of root aerenchyma; and (5) the partitioning of a greater proportion of biomass to the root system. It is possible to breed for this ideotype to develop crops for low-P soils (Lynch, 2011, 2013; Lynch and Wojciechowski, 2015). Efficient genotypes of common bean and maize have shallow roots in the topsoil. Shallower root growth angle of axial or seminal roots increases topsoil foraging and thereby contributes to higher PAE.

In addition to root system architectural traits, root morphological traits such as root length, diameter, surface area and volume, presence of root hairs, and length of root hairs contribute to inter- and intra-specific variation in PAE. The formation of root cortical aerenchyma, which converts living cortical tissue to air space through programmed cell death, improves PAE by reducing the metabolic cost of soil exploration (Lynch and Wojciechowski, 2015). A cost–benefit analysis of root traits indicated that root hairs have the greatest potential for improving PAE relative to their cost of production (Brown *et al.*, 2013). Greater gains in PAE can be achieved through increased length and longevity of root hairs, as compared with increasing their density. The combination of long root hairs and shallow basal roots has a synergistic effect on P acquisition that translates to a three-fold increase in shoot biomass of cultivars with both traits (Miguel *et al.*, 2015). Genetic variation in root hair length can be exploited to develop crop cultivars with improved PAE due to their ability to expand the effective P depletion zone around the root axis (Lynch and Wojciechowski, 2015). Dimorphic root architecture with axial roots with a greater range of growth angles could also be considered for improving PAE of crop plants grown in low P soils.

An increase in PAE by mobilization of P in the rhizosphere can be achieved by increased production and secretion of organic acids and enzymes such as phosphatases and ribonucleases in the rhizosphere (Zhang *et al.*, 2014). However, using near-isogenic lines of wheat, Ryan *et al.* (2014) showed that citrate efflux had no consistent advantage for total biomass or grain yield in multiple field trials on different soil types and different P treatments.

Another means of improving PAE in several crops is through association between plant roots and arbuscular mycorrhizal fungi (Ramaekers *et al.*, 2010; Richardson and Simpson, 2011) although processes independent of P nutrition may also be important (Suriyagoda *et al.*, 2014).

Significant genetic variation in root adaptations to low P soils has been reported in several crops, including rice, maize, common bean and soybean, and numerous quantitative trait loci (QTL) encoding traits for crop PAE have been identified (for reviews see López-Arredondo *et al.*, 2014; Zhang *et al.*, 2014). At a given root system size, up to three-fold variation in whole-plant biomass was found among 196 rice accessions under P deficiency, indicating that genotypes differed in how efficiently their root system acquired P to support overall plant growth (Mori *et al.*, 2016). Conventional breeding showed significant progress in developing P-efficient cultivars, particularly for soybean in China (Wang *et al.*, 2010). But success with marker-assisted breeding has been limited due to significant environmental effects on traits influencing PAE resulting in most QTL identified making small contributions to overall P efficiency. Rice lines showing a dramatic increase in PAE when grown in P-deficient soils were developed using QTL, *Pup1* (*Phosphorus uptake 1*) in marker-assisted breeding (Chin *et al.*, 2011). Overexpression of PSTOL1 – the gene responsible for *Pup1* QTL – increased rice yield on P-deficient soil, indicating potential for further genetic enhancement of P efficiency in rice (Gamuyao *et al.*, 2012). Although very promising experimental results were obtained through transgenic approaches in improving P efficiency in different crops (López-Arredondo *et al.*, 2014; Zhang *et al.*, 2014) there has not yet been a transgenic commercial cultivar produced with improved adaptation to low P soils.

## ROOT ADAPTATIONS TO ALUMINIUM TOXIC SOILS

Approximately 40 % of arable soils worldwide are acidic and rhizotoxicity of Al is the primary limitation to crop and forage yields on most acid soils (Rao *et al.*, 1993; Kochian *et al.*, 2015). Developing crop genotypes tolerant to acid soil conditions is an ecologically friendly, energy-conserving and economical solution for resource-poor farmers in the tropics. Genetic variation exists for acid soil adaptation among crops and genotypes within a crop. These genotypic differences in yield could be related to differences in resistance to Al, and/or acquisition and utilization of nutrients for transport of photoassimilates to developing grain. Field screening for Al resistance would seem to be the most desirable approach, because it best approximates the intended cropping environment (Haling *et al.*, 2011; Yang *et al.*, 2013). In practice, however, reliable ranking of genotypes in the field has been difficult. This is mainly because exchangeable Al levels are not uniform and environmental factors may interact with soil Al to mask the expression of Al resistance. Thus, it is necessary to combine field with greenhouse screening techniques based on physiological traits of Al resistance (Rao, 2014).

Despite the rhizotoxicity of Al being identified over 100 years ago, there is still no consensus regarding the mechanisms whereby root elongation rate is initially reduced. At soil pH values of 5 or below, toxic forms of Al are solubilized and excess levels of toxic Al inhibit root growth and function (Delhaize and Ryan, 1995; Horst *et al.*, 2010). The primary and earliest symptom of Al toxicity is a rapid (within minutes) inhibition of root elongation (Ryan *et al.*, 1993; Sivaguru and Horst, 1998; Kollmeier *et al.*, 2000), and thus crops suffering from Al toxicity are assumed to be at greater risk of drought due to limited root development (Yang *et al.*, 2013). The distal part of the transition zone in the root apex was identified as the primary site of action of toxic Al ions (Sivaguru and Horst, 1998). Callose formation in root apices of maize is an excellent indicator of Al injury (Eticha *et al.*, 2005) and callose content in the root tips of inbred lines could be used as a tool to discriminate and select acid soil-adapted inbred lines, but had limited value for predicting hybrid performance (Narro and Arcos, 2010).

Research conducted over the past two decades on the physiology, genetics and molecular biology of plant Al resistance and toxicity has shown that Al resistance can be achieved by mechanisms that exclude Al from the root apex apoplast (Al exclusion) and/or by mechanisms that enable plants to tolerate Al in the symplast (Al tolerance) (Table 2; Kochian *et al.*, 2004, 2015; Ma, 2007; Ryan and Delhaize, 2012; Yang *et al.*, 2013). Al exclusion is an Al resistance mechanism based particularly on exudation of Al-chelating organic compounds (e.g. organic acids or phenolics) into the rhizosphere, preventing toxic Al species from entering root cells. Al tolerance is an Al resistance mechanism in which Al^3+^ ions are sequestered and detoxified in subcellular compartments and/or translocated away from the root tip apoplast. There is a need to define the precise mechanism of Al-induced inhibition of root elongation. It has been a matter of debate whether the primary lesions of Al toxicity are apoplastic or symplastic (Horst *et al.*, 2010). Although there is evidence for symplastic lesions of Al toxicity, the protection of the root apoplast appears to be a prerequisite for Al resistance in Al-excluder and Al-accumulator plants. Recently, Kopittke *et al.* (2015) examined soybean roots exposed to Al using high-resolution kinematic analyses, molecular biology, rheology and advanced imaging techniques and showed that the primary lesion of Al is apoplastic. They found that 75 μm Al reduced root growth after only 5 min (or 30 min at 30 μm Al), with Al being toxic by binding to the walls of outer cells, which directly inhibited their loosening in the elongation zone. They also found that an alteration in the biosynthesis and distribution of ethylene and auxin was a second, slower effect. Their study demonstrated the importance of focusing on root traits related to cell wall composition as well as mechanisms involved in wall loosening to overcome the deleterious effects of soluble Al.

Kochian *et al.* (2015) reviewed the progress from work on the molecular basis for crop Al resistance and made the following observations: (1) multiple Al resistance genes underlying novel mechanisms have been identified; (2) the best-characterized mechanism of Al resistance, root tip Al exclusion through Al-activated organic acid exudation, involves genes encoding Al-activated malate transporters (ALMTs) and the multidrug and toxin extrusion (MATE) citrate transporters; (3) the molecular basis of both constitutive and Al-induced Al resistance gene expression is beginning to be elucidated, with identification of both *cis*-elements and *trans*-acting factors involved in the expression of several Al-resistance genes; (4) novel Al tolerance genes have been identified involving modifications to the carbohydrate composition of the root cell wall, leading to reduced wall Al accumulation; (5) other Al tolerance mechanisms involve the identification of novel Al uptake transporters, including Nrat1 in rice (which moves Al from the cell wall into root cells, where it is sequestered in the vacuole) and aquaporins (which mediate plasma membrane and tonoplast Al accumulation in an Al accumulator); (6) major Al tolerance loci that have been pivotal in breeding strategies targeting crop adaptation to Al-toxic soils are determined primarily by the plasma membrane transporters conferring Al-activated organic acid release (ALMTs and MATEs); and (7) there is breeding potential in exploring the genetic determinants of transcriptional regulation of ALMT1 and MATE genes in addition to those underlying other Al tolerance mechanisms.

Differential genotypic response to Al stress is the basis of identification of new sources of Al resistance and contributes to improved understanding of the mechanisms of Al resistance. Yang *et al.* (2013) reviewed the progress in defining the mechanisms of Al resistance in common bean using the Al-resistant cultivar ‘ICA Quimbaya’ and the Al-sensitive line ‘VAX-1’. The analysis of spatial growth profiles revealed that the initial inhibition of root elongation by Al resulted from a generalized effect along the entire elongation zone (Rangel *et al.*, 2007). The induced and sustained Al resistance of ‘Quimbaya’ is found to be mediated by reducing the stably bound Al in the apoplast, thus allowing cell elongation and division to resume (Rangel *et al.*, 2009). The kinetics of citrate exudation from root tips offered the most consistent explanation for the response in root elongation and Al uptake of both Al-resistant and Al-sensitive genotypes of common bean to Al treatment (Rangel *et al.*, 2010). Resistance to Al in common bean is attributed to the release of citrate by the root apex, which is mediated by the MATE citrate transporter gene (Eticha *et al.*, 2010). Al resistance was mainly dependent on the capacity to sustain citrate synthesis, thereby maintaining the cytosolic citrate pool that enables exudation. The initial Al-induced inhibition of root elongation in both Al-resistant and Al-sensitive genotypes was correlated with the expression of the 1-aminocyclopropane-1-carboxylic acid oxidase gene (Yang *et al.*, 2013).

The runner bean (*Phaseolus coccineus* L.) germplasm accession G35346 showed high shoot vigour in an acid soil in the field and superior root elongation in Al-toxic soil and hydroponic systems in the greenhouse (Butare *et al.*, 2011). One selection, G35346-3Q, expressed excellent root development in all three evaluation systems, and appeared to offer a resistance mechanism that could be selected readily in any of these systems, and was crossed to the drought-resistant but Al-sensitive common bean line SER 16. A derived line, ALB 91, expressed much of the root vigour of the Al-tolerant parent and has been used extensively in crosses (Butare *et al.*, 2012). However, only very few progenies were similar to G35346-3Q; most expressed one or another trait of the drought-resistant parent (Butare *et al.*, 2012). Resistance to Al appears to be complex in *Phaseolus coccineus* and seems to be the result of a combination of traits that segregated among the progenies. This probably indicates that multiple traits are required to confront an acid soil complex, of which Al resistance is one component. While resistance sources for individual stresses can be employed in breeding, it may be necessary to combine these multiple traits and subject breeding populations to relevant selection pressure under field conditions.

*Brachiaria* grasses are the most widely planted forage grasses in the tropics (Miles *et al.*, 2004). Studies on the mechanisms of Al resistance in *Brachiaria* grasses indicated that well-adapted *Brachiaria decumbens* Stapf (signalgrass) tolerated an approximately five-fold higher level of Al than poorly adapted *Brachiaria ruziziensis* Germain & Evrard (ruzigrass), even though the resistance of ruzigrass was comparable with that of wheat, triticale and maize genotypes that were previously classified as Al-resistant (Wenzl *et al.*, 2001). The very high level of Al resistance found in signalgrass was not associated with secretion of organic acids and phosphate at root apices (Wenzl *et al.*, 2001). A possible role for root plasma membrane (PM) negativity and/or PM composition was observed for the high level of Al resistance in signalgrass (Watanabe *et al.*, 2011). Arroyave *et al.* (2011) found that Al-induced changes in root epidermal cell patterning were a distinctive feature of high-level Al resistance in signalgrass. Al resistance was related to less Al accumulation particularly in root hairs accompanied by an Al-induced increase of chlorogenic acid, indicating a possible role for chlorogenic acid as a primer for changes in root epidermal cell patterning that may contribute to Al hyper-resistance in signalgrass.

Interspecific *Brachiaria* hybrids are developed using *B. decumbens* with high adaptation to low P and Al-toxic acid soils combined with *B. brizantha* (A. Rich.) Stapf (palisadegrass) with its high level of resistance to spittlebug, a major insect pest (Miles *et al.*, 2004). Implementation of a simplified version of a screening method using vegetative propagules (Wenzl *et al.*, 2006) allowed simultaneous assessment of Al resistance and root vigour based on visual inspection and contributed to breeding progress toward improving acid soil adaptation (Wenzl *et al.*, 2006). Fifteen hybrids were identified with superior Al resistance. Field evaluation of 15 brachiariagrass hybrids together with three parents and four checks over 3 years at a location in the Colombian Llanos with infertile acid soil and no maintenance fertilizer resulted in identification of cultivar ‘Mulato II’ and two hybrids that were superior to the others (Rao, 2014). Intraspecific hybrids of *B. humidicola* (Rendle) Schweick (koroniviagrass) are being developed that are not only highly adapted to low-fertility acid soils but are also highly tolerant to poorly drained soils with constitutive formation of RCA in their root systems (Jiménez *et al.*, 2015). In addition, these hybrids also have great potential to reduce nitrification in soil and emission of nitrous oxide to the atmosphere (Rao *et al.*, 2014).

Bulbous canarygrass is one of the most important sown perennial grasses used in south-eastern Australia due to its high productivity and drought tolerance (Culvenor and Simpson, 2014). However, interactions among climate, soil acidity and grazing pressure can affect its persistence. A programme to improve acid soil tolerance resulted first in the release of ‘Landmaster’, and recently ‘Advanced AT’, which is the most Al-tolerant cultivar of bulbous canarygrass, bred by recurrent selection on acid soils in a population containing genes from a related, more Al-tolerant species, *P. arundinacea*. Culvenor and Simpson (2014) indicated that the higher level of Al tolerance observed with ‘Advanced AT’ is of most benefit in its more reliable establishment on acid soils under variable moisture conditions.

Lucerne is the most widely grown forage legume in the world. Its productivity is affected by reduced root growth due to soil acidity and Al toxicity (Khu *et al.*, 2012). Recurrent selection based on field performance has been used to develop acid soil-tolerant germplasm (Georgia-Acid Tolerant). Molecular markers associated with acid soil and Al tolerance were identified (Khu *et al.*, 2013) and Al tolerance from different transgenes has been documented (Reyno *et al.*, 2013). A combination of breeding methods with marker-assisted recurrent selection could accelerate genetic gain for Al tolerance in lucerne (Reyno *et al.*, 2015).

The above comparison of relative differences in Al resistance in different food and forage crops indicates that there is a large gap between the level of Al in soil solution that wheat can tolerate versus the level that forage crops such as bulbous canarygrass or signalgrass can tolerate. Identification of genes responsible for greater level of Al resistance in forage crops can contribute to further improvement of Al resistance in food crops.

## IDEOTYPES FOR IMPROVING ROOT ADAPTATIONS

An ideotype is a plant with an ideal phenotype that combines the desirable traits and mechanisms predicted to enable its adaptation to a target environment. A crop ideotype is a model plant that is expected to yield more when developed as a cultivar (Donald, 1968). Ideotype is also defined as a combination of morphological and/or physiological traits optimizing crop performance to a particular biophysical environment, crop management and end-use (Martre *et al.*, 2015). Root phenes play a major role in improving adaptation to problem soils, and matching the root phenes to specific soil conditions will be a particular research challenge for the future (White *et al.*, 2013*a*). Different root systems will be required for different soil environments.

The SCD root ideotype proposed for efficient N acquisition combines several root phenes (Lynch, 2013; White *et al.*, 2013*a*). These include: early root vigour, large root biomass or root/shoot ratio, more cortical aerenchyma, initial roots with shallow growth angles and later roots with steep root growth angles, larger root surface area, high root length density, greater N uptake capacity of root cells, greater water uptake through enhanced transpiration, greater exudation of biological nitrification inhibitors and greater association with organisms fixing N_2_. The root ideotype for topsoil foraging for P combines several desirable root phenes (White *et al.*, 2013*a*). These include: early root vigour, large root biomass or root/shoot ratio, more cortical aerenchyma, larger root surface area in topsoil, high root length density, proliferation in patches of high P availability, mycorrhizal associations, greater exudation of H^+^ and organic compounds, greater exudation of phosphatases, and greater phosphate uptake capacity of root cells. A shallow root system will be beneficial to maximize P acquisition in a low P soil while deep root system will improve nitrogen and water acquisition, particularly in deep soils (Lynch, 2013). Deep rooting can also be important for P acquisition if P occurs at depth and the topsoil is dried out. This acknowledges that P is generally poorly mobile in soil and can be strongly stratified with the majority of applied P located in the topsoil. There are situations where P moves down the soil profile (e.g. sandy soils) or is located in the subsoil due to the nature of the soil. Greater numbers of root tips will improve Ca uptake while exudation of organic acids will serve as a defence mechanism against Al toxicity or as an efficient mechanism of P acquisition (George *et al.*, 2012; Yang *et al.*, 2013).

An integrated improvement of root adaptations to problem soils is necessary to improve crop productivity on low-fertility acid soils rather than considering individual nutrient stresses separately. Thus, we need a common ideotype with phenotypic plasticity to respond to several constraints simultaneously. The aim of this common ideotype could be not only to improve nutrient and water acquisition and resistance to high Al, but also to optimize the balance between internal use of carbon resources for structural development, and the benefits gained from such investments in terms of the fraction of photosynthates that are translocated to economical products. By definition this should lead to better yield, and is useful only if it leads us to focus on the factors that maximize both biomass production and harvest index.

Low soil fertility and Al toxicity in particular limit biomass accumulation. Based on a modelling exercise, Nord *et al.* (2011) concluded that P acquisition would be favoured by a longer growth cycle. A longer crop cycle translates into more resource acquisition, which is to say, more P, K, Ca and other nutrients (Nord and Lynch, 2009). The rate of root elongation in crops is drastically reduced in the presence of Al toxicity and slower root growth would likewise be compensated for by a longer vegetative phase. On the other hand, a longer vegetative cycle also exposes the crop to risk of drought over a longer time, and augments the expenditure of water from the soil, running the risk of exhausting this resource before the critical grain filling stage. Stomatal control and higher values of transpiration efficiency can ameliorate this risk (Sinclair, 2012). Regarding resource acquisition from the soil, particularly for P, at least two root phenes appear to be valuable. The anatomical phene of long, dense root hairs and the architectural phene of shallower basal root growth angle are synergistic for P acquisition (Miguel *et al.*, 2015). Another root phene with wide utility could be greater specific root length, although this strategy might have limitations in compacted or heavy textured soils if roots with a smaller diameter have a diminished ability to penetrate the soil (Butare *et al.*, 2012).

Multiple soil stress factors often co-occur in farmers’ fields. Roots that are stunted by Al toxicity are inefficient in absorbing both nutrients and water and Al-resistant plants may be more drought-resistant and require lower inputs of lime and P fertilizer than less resistant genotypes (Yang *et al.*, 2013). Tang *et al.* (2002) showed that Al-tolerant wheat grew roots deeper and used more water than Al-sensitive wheat in an acid soil in the field. On many low-fertility or acid soils of the tropics, variability in rainfall distribution and longer dry spells during the main growing period of crops are becoming increasingly important yield-limiting factors with global climate change. As a result, crop plants are needed that combine root adaptations to soils with low fertility and toxicities with adaptation to drought (Yang *et al.*, 2013).

**Fig. 1.**
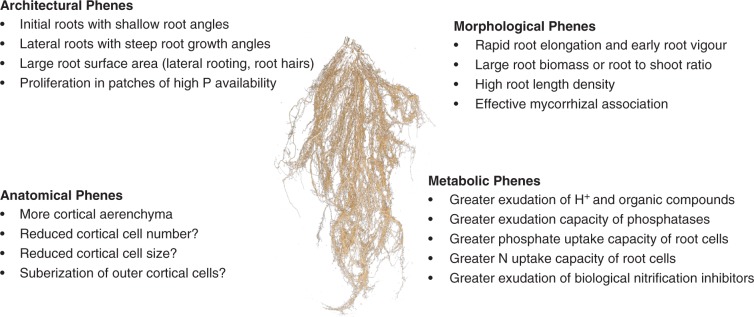
A desirable combination of root phenes for an ideotype of a crop or forage cultivar for improved adaptation to low fertility and aluminum-toxic soil conditions. The root image is from *Brachiaria humidicola* ‘Tully’ (Photo: J. A. Cardoso).

Interactions of a particular stress with other factors in the environment, especially other stresses, complicate the selection for stress tolerance (Lynch, 2013; Yang *et al.*, 2013; Beebe *et al.*, 2014). Identifying the critical interactions and incorporating these into a selection programme is perhaps the most challenging aspect of improving adaptation to soils with low fertility and toxicities. An ideotype suggested for improved yield in soils with low fertility and Al toxicity must combine root and shoot traits both for increased resource acquisition and biomass accumulation, and for enhanced partitioning of biomass to grain or the harvested product. A dimorphic root system that combines shallow roots with deep roots may be more suited for improving crop and forage adaptation to soils with low fertility and Al toxicity. A general framework suggested for this would be rapid development of a shallow root system with Al resistance that favours P acquisition for early canopy development combined with development of a deep root system that favours N acquisition from deeper soil layers and could also improve adaptation to seasonal drought (Fig. 1). While this ideotype could be useful under a range of growing environments, it would be especially relevant to address the limitations of low-fertility soils that are prevalent in the smallholder farms of the tropics. Such an ideotype with phenotypic plasticity could require combining a number of root architectural, morphological, anatomical and metabolic traits through conventional and molecular breeding approaches.

## CONCLUSIONS AND FUTURE PERSPECTIVES

This review shows that considerable progress has been made in the last decade in improving knowledge of root adaptations to soils with low fertility and Al toxicity. Developing the right root system to cope with soil fertility and toxicity constraints in each production environment will be a major research challenge.

Low N-adapted maize genotypes have few crown roots and acquire more N from deep soil strata. These genotypes also have the ability to form high RCA to improve plant growth under low-N conditions by decreasing root metabolic costs, thereby enhancing soil exploration and N acquisition from deep soil strata.

Phosphorus acquisition efficient common bean genotypes develop more adventitious roots, shallower basal roots, and longer, denser root hairs. Traits including root hair length, adventitious rooting and basal root growth angle under low P availability were shown to be under control of multiple QTL. Architectural trade-offs for P and water acquisition have been demonstrated for root growth angle. Genotypes with deeper basal roots had superior growth under water stress while genotypes with shallower basal roots have superior growth under low P conditions. Rice lines developed using QTL, *Pup1* showed a dramatic increase in PAE when grown in P-deficient soils.

Significant advances have been made in defining the mechanisms of Al resistance in different crops and forages using Al-resistant and Al-sensitive cultivars. Major Al tolerance loci are determined primarily by the plasma membrane transporters conferring Al-activated organic acid release.

The genetic control of stress tolerance is often complex, requiring a combination of several different mechanisms to achieve significantly elevated levels of stress tolerance. In these cases, gene discovery and efficient marker-assisted gene pyramiding technologies will be important.

Physiological and molecular studies on breeding populations provide a path towards the identification of physiological mechanisms and genomic regions contributing to root system responses to low soil fertility and Al toxicity, which in turn could lead to the isolation of genes contributing to changes in individual root phenes. Major advances have been made in developing screening methods at laboratory, greenhouse and field level to evaluate differences in root phenes for tolerance to N and P deficiency and Al resistance. Using these screening methods, it is possible to make further advances in breeding or agronomic evaluation of crops and forages for improved root adaptation to soil fertility constraints. Advanced root phenotyping tools will address major knowledge gaps to dissect the root responses into specific root phenes that will aid breeders to develop superior crop and forage cultivars. These new cultivars will play a key role in sustainable intensification of crop–livestock systems, particularly in developing countries. Development of these new cultivars is essential to improve global food and nutritional security.

A major challenge is to define the interactions between different soil constraints and their influence on root adaptations so that cultivars with adaptation to soils with low fertility and Al toxicity can be developed through breeding. Dissection of differences in individual root phenes as influenced by mineral element deficiency or toxicity could explain differences in field performance between genotypes. The key to detecting subtle changes in growth is to be familiar with whole plant development and plasticity responses to stress conditions.

Combining a number of root architectural, morphological, anatomical and metabolic phenes for improved adaptation to soils with low fertility and Al toxicity will require multidisciplinary approaches. It is critical to cross-validate and integrate information from breeding, agronomy, physiology, soil science, plant nutrition and molecular genetics. Collaboration between breeders and physiologists contributes to defining appropriate root phenes that can serve as selection criteria for breeding, and to help design selection schemes and methods to address major soil constraints (Beebe, 2012; White *et al.*, 2013*a*, *b*; Wishart *et al.*, 2013; Yang *et al.*, 2013; Rao, 2014; Lynch, 2015; Lynch and Wojciechowski, 2015). Experts in genomics, transgenics and bioinformatics contribute to define which genes and mechanisms are most promising so that an aggressive breeding programme will be able to improve adaptation to soils with low fertility and Al toxicity. The combination of multiple complementary strategies should be an integral part of crop improvement and is expected to enable researchers and breeders to more efficiently address the current and future demands of modern (smallholder as well as industrial) agriculture and food production presently exacerbated by global climate change (Lynch, 2015; Araújo *et al.*, 2015).

Major technological advances have been made in recording images of root systems and for characterizing root phenes under laboratory (Rellán-Álvarez *et al.*, 2015), laboratory and greenhouse (Mairhofer *et al.*, 2013; Le Marie *et al.*, 2014; Richard *et al.*, 2015) and field conditions (Trachsel *et al.*, 2011; Bucksch *et al.*, 2014) (see review by Paez-Garcia *et al.*, 2015). Recently, the Root System Markup Language format was developed to facilitate sharing of root architectural data among different software packages and to provide a standard format (Lobet *et al.*, 2015). Development of non-intrusive methods to dynamically study root architecture and root distribution *in vivo* will help to design cultivars with optimum root systems for soils with low fertility and Al toxicity. Field studies of mapping populations or association panels are needed to identify key root traits and underlying genes that are able to enhance nutrient acquisition beyond the level present in current cultivars.

A broader view is needed to implement an integrated multidisciplinary approach to make progress in breeding for improved root adaptations for stress resistance. An integrated improvement of resistance to different stresses is likely to be more productive than considering them in isolation (Yang *et al.*, 2013). One aim is to determine trade-offs in investment of carbon between roots versus economic plant parts to strike a balance between internal use of carbon resources within the plant and the benefits gained from such investments in terms of the fraction of photosynthate that can be efficiently translocated to developing roots and economically important products. A challenge is to identify QTL of major effect that are independent of the particular genetic background and to clone the genes in the QTL. The ability of next-generation sequencing and advanced metabolic profiling to co-sequence or co-screen a large number of F_2_ or RILs coupled with statistical linkage analysis could improve the efficiency of molecular breeding for improved root level adaptation to stress factors.

Recent advances in translational genomics and the development of molecular tools for breeders have revolutionized plant improvement strategies by integrating complex biological data to inform genomics-assisted breeding approaches (Wang *et al.*, 2013; Varshney *et al.*, 2015). A combination of genomic selection and genome-wide association studies and mapping populations will improve our ability to connect phenotypes and genotypes, and genomic selection can take advantage of these data for rapid selection and breeding (Morrell *et al.*, 2012; Kole *et al.*, 2015). The socioeconomic impact of improved root-level adaptations of crop and forage genotypes to soil stress factors would be immense in terms of increased food production, more efficient use of purchased inputs, and improved integration of crop–livestock systems benefiting both agriculture and the environment.
